# Supplementary material for: Botrytis cinerea infection accelerates ripening and cell wall disassembly to promote disease in tomato fruit
Source: Plant Physiol. 2022 Sep 2;191(1):575–90. doi: 10.1093/plphys/kiac408 (PMC9806607; doi:10.1093/plphys/kiac408)
Supplement: kiac408_Supplementary_Data [file kiac408_supplementary_data.zip › kiac408_Supplementary_Data/Supplemental_Figures_S1S4.pdf]

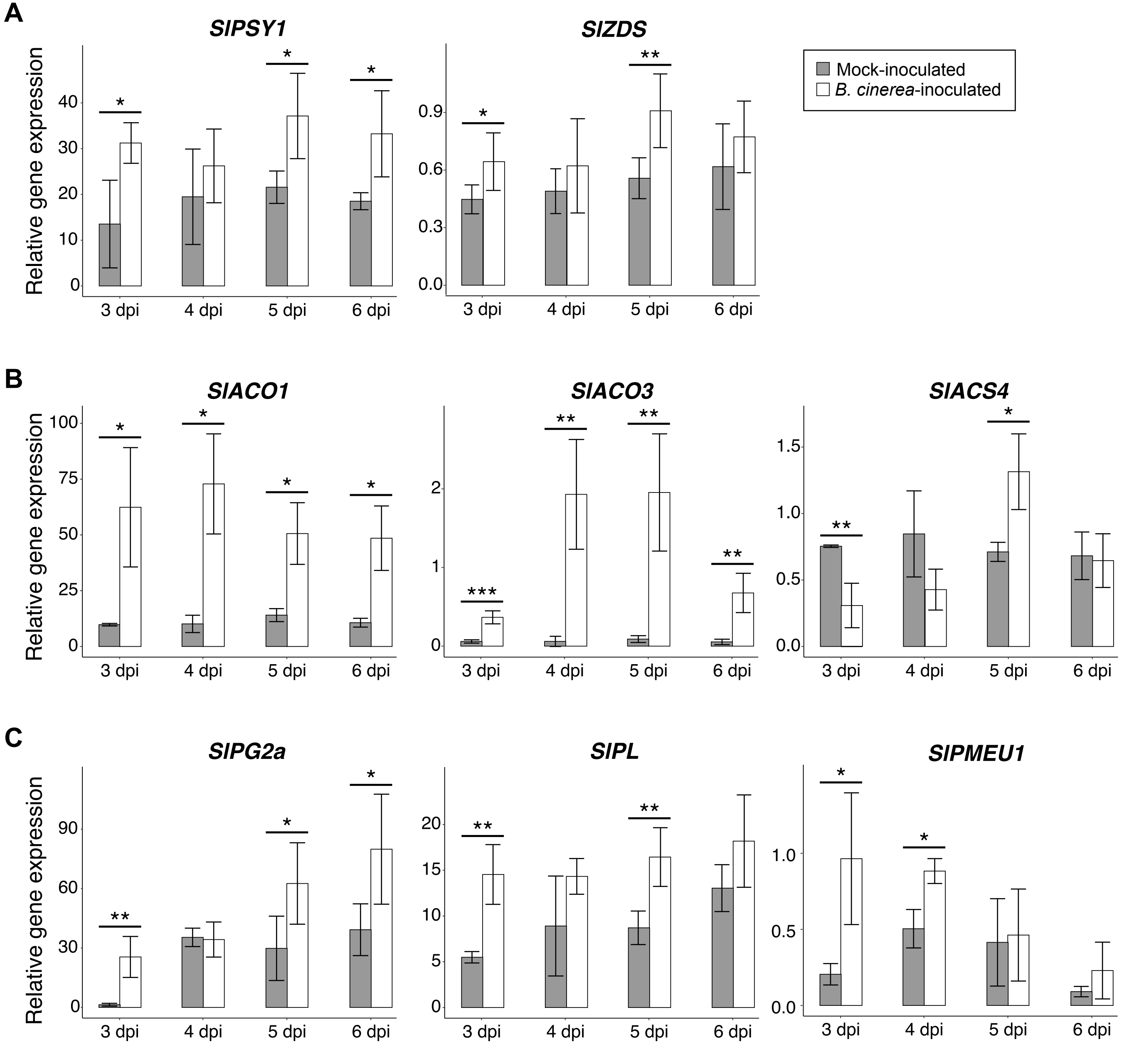

Supplemental Figure 1: qPCR-based expression of selected tomato ripening-associated genes after inoculation with *B. cinerea*. Names of each gene are given above each graph. Asterisks indicate statistical differences (*, $P < 0.05$; **, $P < 0.01$; ***, $P < 0.001$) between mock-inoculated and *B. cinerea*-inoculated Mature Green (MG) fruit across 3 to 6 days post-inoculation (dpi), as calculated by t-test (n = 3 - 8). Error bars correspond to the standard error of the mean. **(A)** Tomato carotenoid biosynthesis genes: phytoene synthase (*SlPSY1*) and zeta-carotene desaturase (*SlZDS*). **(B)** Tomato ethylene biosynthesis genes: 1-aminocyclopropane-1-carboxylic acid oxidases (*SlACO1, SlACO3*) and 1-aminocyclopropane-1-carboxylic acid synthase (*SlACS4*). **(C)** Tomato cell wall degrading enzymes: polygalacturonase (*SlPG2a*), pectate lyase (*SlPL*), and a pectin methylesterase (*SlPMEU1*).

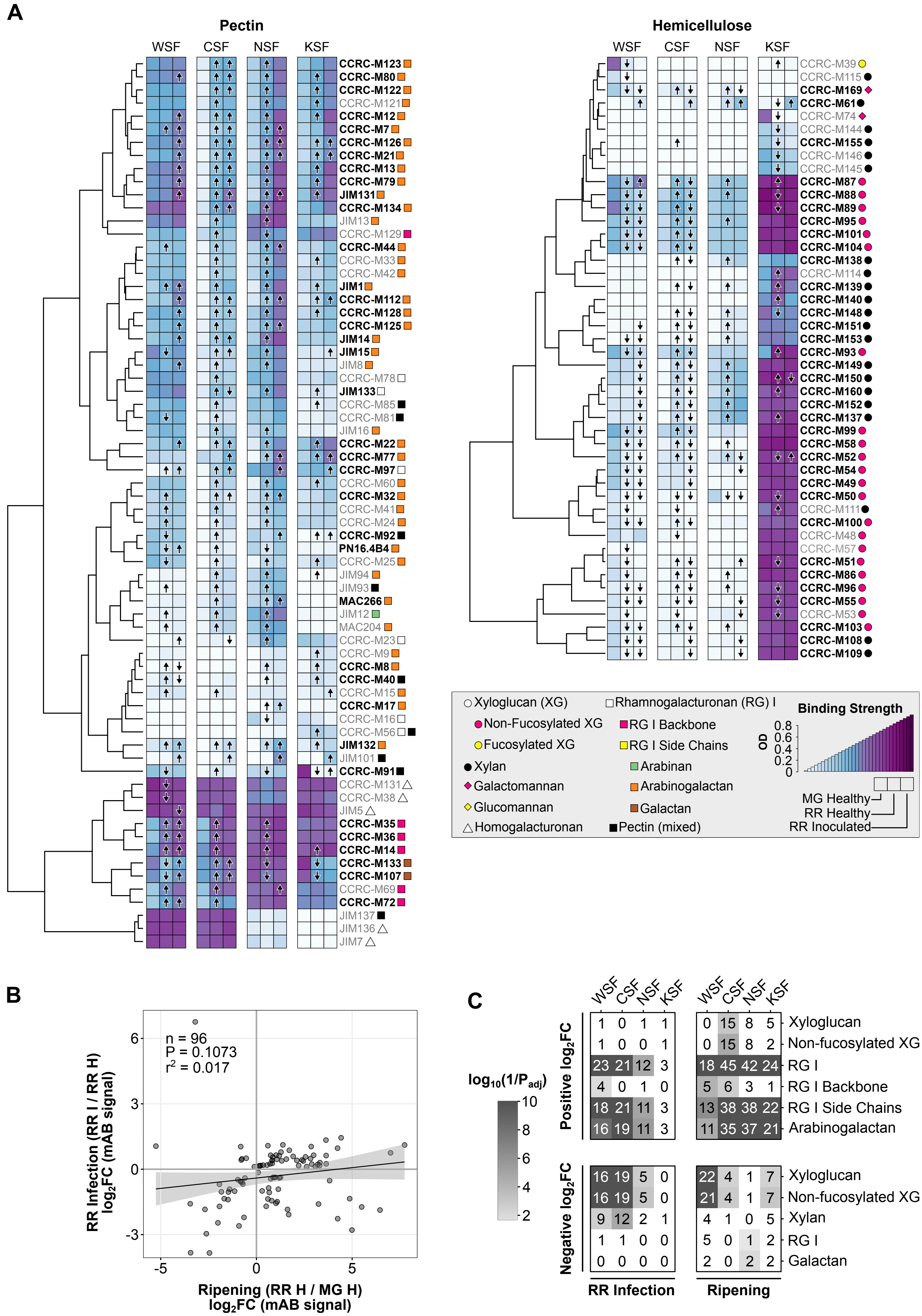

Supplemental Figure S2: Glycomics profiling of *B. cinerea*-inoculated ripe fruit. (A) Heatmaps of binding strength of polysaccharide-binding mABs measured as optical density (OD) in healthy Mature Green (MG H), *B. cinerea*-inoculated Red Ripe fruit (RR I), and healthy Red Ripe (RR H) at 3 days post-inoculation (dpi) or days post-harvest (dph). mAB codes are given to the right of each heatmap row, with the recognized classes of cell wall polysaccharides indicated by colored shapes according to the given key. mAB listed in boldtype are those included in the scatterplot in panel **B**. Arrows within heatmap tiles indicate statistically significant ($Padj < 0.05$) increasing or decreasing antibody strength when compared via t-test to values in healthy RR fruit (n = 6). **(B)** Scatterplot and linear regression model of $\log_2$ fold change ($\log_2$FC) values of mAB signals in RR inoculation (RR I/ RR H) and ripening (RR H / MG H) comparisons. **(C)** Enrichment of polysaccharide classes with statistically significant positive or negative $\log_2$FCs in each cell wall fraction for the ripening and RR inoculation comparisons. Numbers within each tile indicate the number of mABs with a statistically significant $\log_2$FC in that respective fraction and polysaccharide class. WSF = water-soluble fraction, CSF = CDTA-soluble fraction, NSF = Na_2CO_3-soluble fraction, KSF = KOH-soluble fraction.

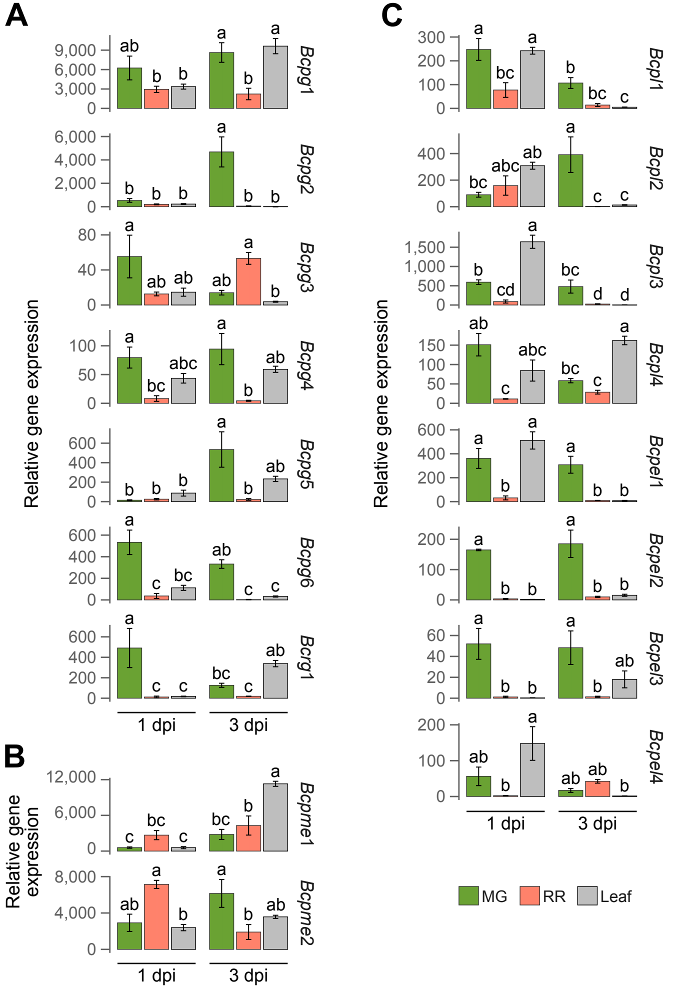

Supplemental Figure S3: RT-qPCR-based expression of selected cell wall degrading enzymes expressed by *B. cinerea* during tomato infections. Names of each gene are given to the right of each graph. Letters indicate statistical differences ($P < 0.05$) between tissues across both 1 and 3 days post-inoculation as calculated by ANOVA and Tukey's HSD test ($n = 4 - 6$). Error bars correspond to the standard error of the mean. **(A)** *B. cinerea* polygacturonase (*Bcpg*) and rhamnogalacturonase (*Bcrg*) genes. **(B)** *B. cinerea* pectin methylesterase (*Bcpme*) genes. **(C)** *B. cinerea* pectin lyase (*Bcpl*) and pectate lyase (*Bcpel*).

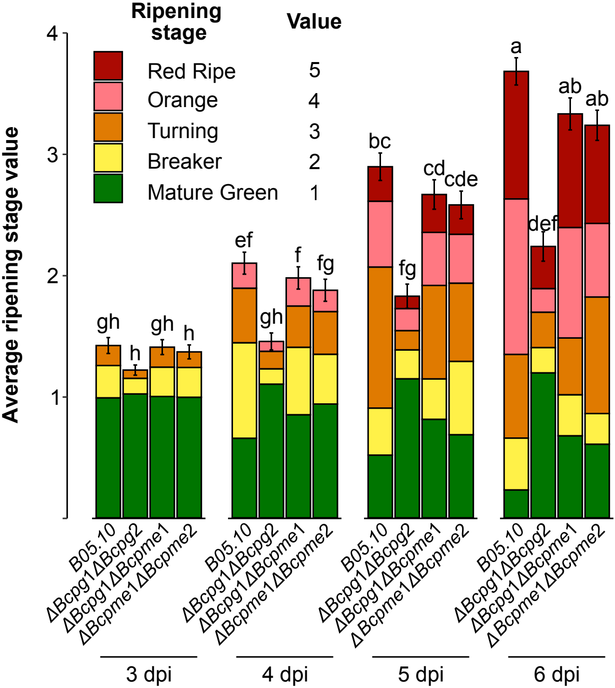

Supplemental Figure S4: Color progression in *B. cinerea* mutant-inoculated Mature Green fruit. Average ripening stage value as assessed by color in B05.10-, *ΔBcpg1ΔBcpg2*-, *ΔBcpg1ΔBcpme1*-, and *ΔBcpme1ΔBcpme2*-inoculated Mature Green fruit (n = 55 - 128). Colored blocks within each column represent the proportion of fruit at that respective stage. Letters indicate statistical differences ($P < 0.05$) between each treatment across all days post-inoculation (dpi) as calculated by ANOVA and Tukey's HSD test. Error bars correspond to the standard error of the mean.
